# The play fever: individual and group factors influence the contagion of play behaviour within social hominid groups

**DOI:** 10.1098/rstb.2025.0144

**Published:** 2026-09-24

**Authors:** Ivan Norscia, Edoardo Collarini, Giada Cordoni

**Affiliations:** Department of Life Sciences and Systems Biology, University of Torino, Torino, Italy

**Keywords:** motor replication, empathy, behavioural contagion, hominids, apes

## Abstract

Via the perception–action model and mirror neuron system, individuals replicate others’ behaviour (behavioural/social contagion) and reduce prediction errors on other behaviours. Among contagious behaviours, play offers a safe context for cooperation, competition and tolerance. Thus, play contagion is interesting to better understand the evolution of social dynamics in many non-primates and primates, including humans. To our knowledge, no study has examined play contagion across social hominids nor analysed individual/group features to identify common modulators. In 2022–2025, we gathered all-occurrences data on play, affiliative and agonistic patterns in social hominids (two groups of captive chimpanzees, bonobos and gorillas; and two toddler classes of ≤ 3 years and their educators). In the 3 min period, we checked if individuals not involved in play would start playing either after the onset of a trigger play session (post-play (PP)) or in absence of any previous play session (matched-control). Play occurrence was highest during PP, informing play contagion. Contagion increased with higher male-to-female, immature-to-adult and affiliative-to-agonistic interaction ratios. Males, youngsters and individuals that were in trigger proximity (≤ 10 m) showed the highest contagion. Shared across social hominids, play contagion and its common modulators might trace back to their last common ancestor, underlying the transmission of behaviour, and possible underlying emotions, within groups.

This article is part of the theme issue ‘Mechanisms, development, phylogeny and functions of emotional expressions’.

## 1. Introduction

Behavioural contagion, a form of behavioural matching, occurs when an individual performs a species-typical behaviour after perceiving another individual performing a similar one [1,2]. Behavioural contagion can involve both self-directed and interactive complex behaviours (e.g. scratching, yawning, grooming, play and aggression [3–8]), rather than single-component actions and typically unfolds over variable time windows, from seconds to minutes [2,9–12].

Behavioural contagion represents an automatic motor replication process through which one individual's behaviour induces a similar output in another [13,14]. In behavioural contagion, the observed behaviour may act as a matching social releaser, eliciting the same type of behaviour, though not necessarily the same motor sequence [12]. This differs from automatic mimicry, in which a more precise behavioural mirroring is observed, possibly via mirroring social releasers [8,12].

At its core, behavioural contagion relies on involuntary perception–action coupling mechanisms [9,15], which have been linked to the mirror neuron system (MNS) and the perception–action model (PAM). MNS is a neural mechanism through which the observation of another individual's action activates corresponding motor representations in the observer. In this way, action perception is directly linked to action coding, so that observing a behaviour can prime the observer to produce a similar response. Within this framework, what is especially relevant is not merely the visible movement itself but the goal-directed meaning of the observed action or of an action-pattern sequence [15,16]. PAM explains how perceiving another's behaviour can automatically activate corresponding representations in the observer, thereby facilitating matched behavioural responses. In PAM, the central aspect is that the observer's response is not treated as a simple mechanical copy of the perceived action but as a process shaped by the observer's prior experience, which modulates how perception is translated into action [14,17].

Behavioural contagion, as a form of motor replication *sensu lato*, may have played an important role in the evolution of human social dynamics, as it can allow individuals to reduce the prediction error concerning others’ behaviours, thereby influencing the continuation or interruption of social interactions [18].

Although the available literature is limited and mostly biased on studies on yawn contagion, there is sufficient evidence showing that the modulation of behavioural contagion depends on a combination of individual and dyadic factors—such as sex, age, social closeness and spatial proximity—which shape how contagion manifests across species and groups [2]. Regarding individual factors, sex-related effects on behavioural contagion are variable and may mostly involve the dominant sex (e.g. yawn contagion in chimpanzees, *Pan troglodytes*; bonobos, *Pan paniscus* [2,7,19–21]). Previous studies indicate that age can modulate behavioural contagion: in some cases, juveniles are more responsive (e.g. aggression contagion in chimpanzees; [7]), whereas in others, adults show higher susceptibility (e.g. yawn contagion in chimpanzees; [22]). Regarding dyadic factors, yawn contagion can be enhanced by affiliation and familiarity in humans and other primates (e.g. in red-capped mangabeys, *Cercocebus torquatus* [23]; chimpanzees and humans, *Homo sapiens* [24–26]; but see black-and-white and red ruffed lemurs, *Varecia variegata* and *Varecia rubra* [27]; in bonobos [19,21,28]). In a cohort of toddlers, social closeness showed a reversed effect on yawn contagion, resulting in reduced levels [8]. This variability suggests that the relationship between behavioural contagion and affiliation is contingent on social dynamics and context dependent [2]. Furthermore, spatial proximity may facilitate contagion (e.g. yawn contagion in the lemur *Indri indri* [29]), because the perception of the triggering stimulus is crucial for activating the perception–action process [14]: the closer the stimulus, the more likely it is to perceive it and to elicit a response. Overall, although individual and dyadic factors can modulate contagion, current evidence remains limited in function and behavioural diversity.

Behavioural contagion, also described as social contagion, is not limited to individual or dyadic dynamics but unfolds at a group level [12]. Consistently, it has been defined as a process through which the behaviour of one individual or group elicits similar behavioural responses in another individual or group [2,30]. Empirical evidence shows that contagion can occur both within and across groups—for example in yawn contagion among geladas (*Theropithecus gelada* [31]) or in aggression contagion among chimpanzees [4]. Moreover, in lowland gorillas (*Gorilla gorilla*), threat displays have been described as contagious [32]. Behavioural contagion can be influenced by group-level variables, such as the number of individuals involved (e.g. human applause [33]) and the relational properties of the group (human behavioural choices [34]). In this regard, the modulation of contagion can depend on group features (e.g. social organization and/or affiliation dynamics [2,11,35]). Consistent with the view that behavioural contagion extends beyond simple pairwise interactions [12], research in both human and non-human primates indicates that it fosters behavioural alignment, synchrony and coordination across multiple individuals, thereby contributing to group cohesion and collective regulation (e.g. human work teams and primate social groups [11,36]; yawn contagion in lions, *Panthera leo* [37]). Again, the effect of group-level features is under-investigated.

Play behaviour has been described as contagious in social birds and mammals, with acoustic and visual cues capable of eliciting play, although quantitative evidence remains limited [6,7,38–44]. In behavioural contagion, including play contagion, what is replicated is the same type of behaviour, but the pattern can vary considerably (e.g. play fighting can elicit solitary play [6]). A play session can act as a behavioural cue that facilitates the initiation of play by others not involved in play [12]. Play contagion may have an adaptive value, as play can reduce anxiety in socially tense contexts in the short term [25,45,46] and be related to physical, cognitive and emotional skills in the long term [47–50]. In many social species, play also enhances relationship quality and/or allows competition while reducing the risk of harm [51–55]. The sharing of a playful mood between closely bonded individuals suggests a mechanism of emotional attunement, through which play contagion strengthens affiliative ties [6]. Moreover, spatial proximity and affiliation levels can enhance play contagion,e.g. in African elephants (*Loxodonta africana* [6]). In this species, age and sex may have an effect on play contagion as males may use play more often to compete in a non-aggressive way and because play is age dependent, mostly occurring at young age [55]. For example, play contagion was found to be highest in younger individuals in chimpanzees [7] and African elephants [6]. There are no studies addressing the effect of group-level demographic or social dynamics variables on play contagion although we can hypothesize that these features, also depending on age, sex and social affiliation of group mates, affect play contagion as they affect the contagion of other behaviours.

Play behaviour is a distinct behavioural system [56] and possibly a neural exercise that offers individuals a safe context to interact by oscillating between competition and cooperation in a non-aggressive way, and it has been crucial in the evolution of social dynamics—including in human groups—and of tolerance [55,57–59]. The propagation of play behaviour may, therefore, have contributed to this process.

Based on the available evidence, all social hominids—lowland gorillas, chimpanzees, bonobos and humans—appear to show behavioural contagion, and even in species in which play contagion has not yet been demonstrated, there is potential for it. Orangutans also have this potential, as their behavioural flexibility is high, and recent studies have highlighted a crucial role of mother–offspring social learning in their development. In captivity, they also seem to adopt social behavioural strategies to cope with crowded environments (see the conflict-resolution study by Kopp & Liebal [60]). In this study, we investigate, to our knowledge for the first time, play contagion across hominid species using data collected under naturalistic conditions over several years and through consistent methodologies. We focus on group-living hominids so that we can test not only the individual and dyadic variables that may influence the phenomenon but also group-level factors, which have been largely neglected so far.

Based on the above framework, we formulated the following predictions:

(i)* prediction 1: presence of play contagion.*

Play is a contagious behaviour in different mammalian species (e.g. from rodents to humans [9,39,40]). Contagious behaviour has been observed in humans (e.g. yawn contagion and scratching [3,61]), chimpanzees (yawn contagion, aggression, grooming and play [4,7,62]), bonobos (e.g. yawn contagion [19,21,28]), and possibly lowland gorillas (threat displays [32]). Therefore, we expected to find play contagion across all the social hominid species considered in this study, although we had not enough elements to predict variability across species;

(ii)* prediction 2: effect of play session features.*

Play contagion can be elicited at different time windows, spanning seconds to minutes from the beginning of the triggering play session [6,7]. Both solitary and social play can elicit contagion of different types of play [9,44]. On the one hand, interactive play contains social elements that may facilitate contagion [6,7,39]. On the other hand, social elements do not necessarily induce play contagion, and solitary play in apes is frequently used to induce a playful mood in others [40]. Therefore, although no study has so far addressed the effect of play session features on play contagion, we expected that such features (i.e. social vs solitary play and session duration) would have no effect;

(iii)* prediction 3: effect of individual and dyadic factors on play contagion.*

In human and non-human primates, sex, age and the type of social interactions have been found to affect behavioural contagion, though not in a consistent way (e.g. yawn contagion, humans, chimpanzees and bonobos [2,21,22,28,63–65]). As said above, play is more frequent in non-mature individuals, and play contagion can be highest in juveniles (e.g. in chimpanzees [7]; African elephants [6]).

Moreover, sex may affect play, with immature males playing more, either to compete in a non-aggressive manner or for motor training (e.g. in rats (*Rattus norvegicus*), African elephants, mountain gorillas (*Gorilla beringei*), chimpanzees, bonobos and humans [6,39,66–69]). Higher play frequencies may be linked to higher play contagion, and individuals that play more may be more likely to be induced into play via contagion [6,39]. In this light, we predicted that play contagion would be highest in immature individuals and males (individual factors; *prediction 3a*).

Behavioural contagion is triggered—via perception–action model—by the observation of the triggering stimulus, which can be enhanced by social proximity (e.g. yawn contagion in lemurs, *I. indri* [29]). Consistently, distance can enhance play contagion (as observed in African elephants [6]). With this state of the art, we predicted that play contagion would be highest in individuals with the highest affiliation rates and within close spatial proximity to the triggering individuals (dyadic factors; *prediction 3b*).

(iv)* Prediction 4: effect of group features on play contagion.*

Behavioural contagion can spread across dyads and extend to networks of group members (group identity) [4,8,70,71]. This also occurs in play contagion, which may give rise to contagion networks (e.g. in African elephants [6]). Play behaviour occurs most frequently in immature individuals [38,47,50,57], and play contagion can be highest in these individuals (e.g. in chimpanzees [7]; African elephants [6]). Moreover, in certain species and circumstances, males may play more to reduce aggressive competition (e.g. bonobos, African elephants and humans [6,66,68]), thus making contagion more probable (group demography). Additionally, affiliation rates and affiliation-aggression balance can affect play contagion (e.g. in African elephants [6,12]) although investigation in this respect is meagre (group social dynamics). No study has specifically investigated the effect of group variables on play contagion. However, based on the above information, we expected that play contagion rates would differ across groups (group identity; *prediction 4a*) and would increase as group size increased and with higher male–female and juvenile–adult ratios (group demography; *prediction 4b*), as well as with a higher affiliation–agonistic interaction ratio (group social dynamics; *prediction 4c*).

## 2. Methods

### (a) Study species, groups and data collection

**Figure 1.**
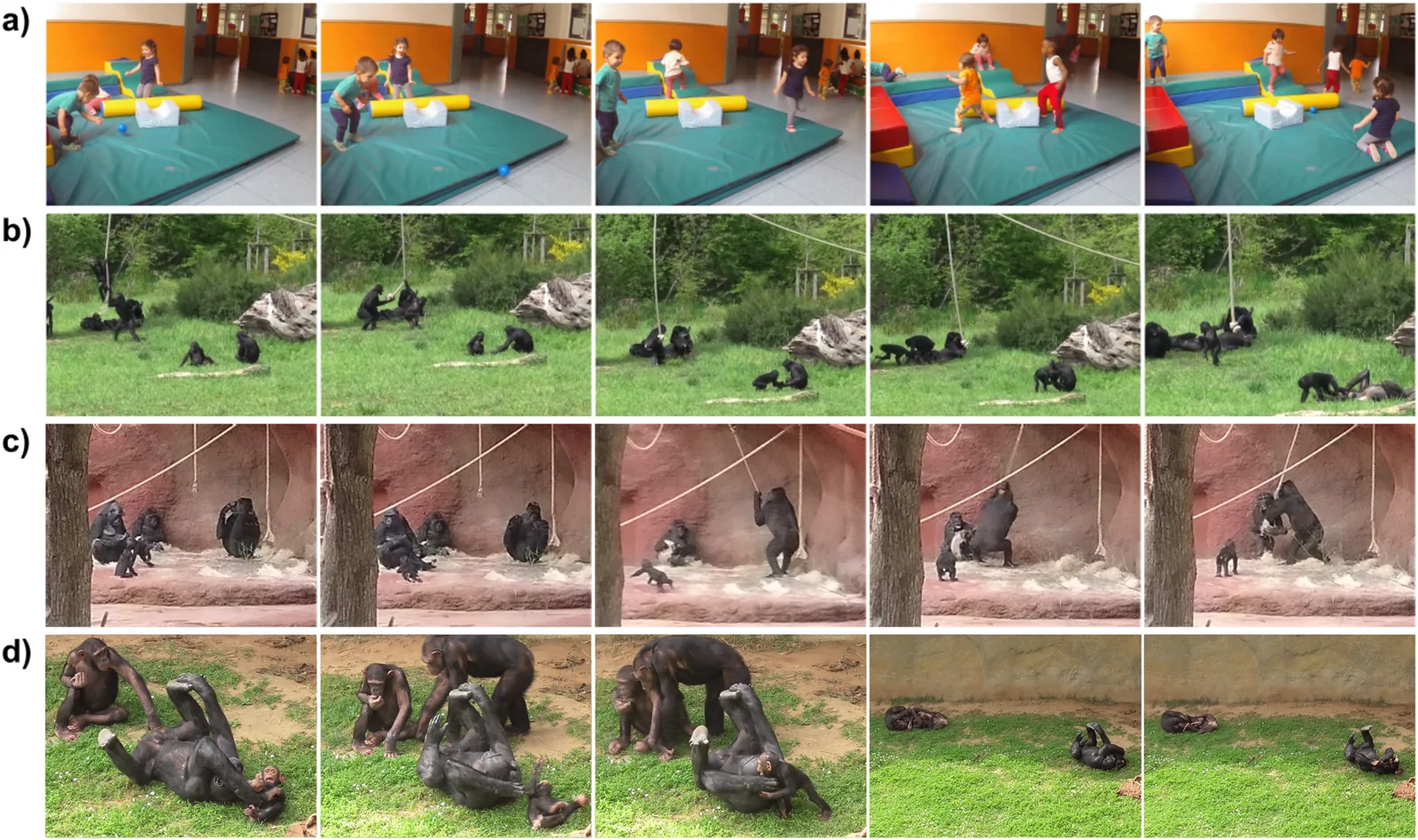
The image sequences show, from left to right in each stripe, (a) humans (top): two toddlers engaging in a social play session, followed by two others starting a new play session; (b) bonobos (second stripe): two juveniles engaging in social play with a rope, followed by a juvenile and an adult initiating a separate play session; (c) gorillas (third stripe): two immatures engaging in social play, followed by two young adults starting a new play session in the same area; and (d) chimpanzees (bottom): an immature and a young adult engaging in social play, subsequently followed by two adults engaging in a new social play session. Sequences extracted from the supplementary material, VIDEO_S1–VIDEO_S4.

From February 2022 to February 2025, we collected behavioural data on humans (*H. sapiens*; young children and adult educators; figure 1a), captive bonobos (*P. paniscus*; figure 1b), lowland gorillas (*G. gorilla*; figure 1c) and chimpanzees (*P. troglodytes*; figure 1d; supplementary material, VIDEO_S1–VIDEO_S4) with two groups studied for each species (humans: HUM_1: *n* = 21, HUM_2: *n* = 14; bonobos: BONO_1: *n* = 15; BONO_2: *n* = 20; gorillas: GOR_1: *n* = 11; GOR_2: *n* = 10; chimpanzees, CHIMP_1: *n* = 7; CHIMP_2: *n* = 9; table 1 shows group composition, data collection period and location) through daily observations via all-occurrences sampling [72]. Data were collected five days per week, from 8.00–9.30 to around 16.00–17.30, either continuously or divided into half-day blocks, for 138.39 ± 23.75 h (group mean ± s.e.). Based on literature, chimpanzees and gorillas were considered subadult–adult (as opposed to immature subjects) over the age of six (with weaning occurring up to 6 years old [73]) and gorillas (weaning up to 5.7 years old [74]). Bonobos, showing longer weaning period, were considered subadults–adults over the age of seven (weaning can last until 6.6–7 years old [75]). Regarding great apes, all zoological facilities included indoor and outdoor spaces (indoor spaces: 235.00  ±  55.90 m^2^, outdoor spaces: 3016.7  ±  484.48 m^2^; mean ± s.d.), and they were similarly enriched with climbing structures, vegetation and objects. Apes had free access to both areas between approximately 21.30 and 17.30, except during adverse weather conditions.

**Table 1.** Overview of the study groups: size, composition and observational sampling.

species	group	location	*n* individuals	observation hours	observation period
**bonobos**	BONO_1	Ouwehands Dierenpark, NL	15 (females: 6 adults, 2 immatures; males: 4 adults, 3 immatures)	121.8	Mar–May 2024
	BONO_2	La Vallée des Singes, FR	20 (females: 7 adults, 5 immatures; males: 7 adults, 1 immature)	73.2	Mar–May 2024
**chimpanzees**	CHIMP_1	Zoo de La Palmyre, FR	7 (females: 4 adults; males: 2 adults, 1 immature)	67.7	Nov 2024–Feb 2025
	CHIMP_2	Zoo de La Palmyre, FR	9 (females: 5 adults, 1 immature; males: 3 adults, 1 immature)	68.8	Nov 2024–Feb 2025
**gorillas**	GOR_1	ZooParc de Beauval, FR	11 (females: 5 adults; males: 4 adults, 2 immatures)	239.7	Feb–Jun 2024
	GOR_2	La Vallée des Singes, FR	10 (females: 4 adults, 1 immature; males: 4 adults, 1 immature)	158.7	Mar–Jun 2024
**humans**	HUM_1	Nursery ‘Armando Melis’, IT	21 (females: 3 adults, 8 immatures; males: 10 immatures)	216.0	Feb–May 2022
	HUM_2	Nursery ‘Armando Melis’, IT	14 (females: 3 adults, 4 immatures; males: 1 adult, 6 immatures)	161.2	Feb–May 2022

Data on young children were collected at the ‘Armando Melis’ nursery in Turin (Italy). The study sample primarily consisted of children in the 13–36 months age range. Exceptions included two female infants (initially aged 10–12 months) who became toddlers early in the study and three late toddlers (two males and one female) who turned older than 36 months during the study period, thus technically becoming pre-schoolers. Here, all young children will be referred to as toddlers. All had been together for at least five months. Adults (educators, one man, six women; age ≥ 25 years old) were considered part of the group and interacted freely with the toddlers. Each classroom had access to an activity room, a shared ‘water room’ and a garden used in rotation.

We collected data on dyadic social play, affiliative interactions and agonistic events (see table 2 for the ethogram, elaborated based on previous literature [54,76–88]; supplementary material, table S1 for more details). Observations were audio- and/or video recorded using full HD devices (ZOOM-Q8, Panasonic HC-V380; Panasonic Lumix DMC-FZ60). A trainer coder (I.N. or G.C.) and each video coder (one per group) achieved Cohen's *κ* interobserver reliability, calculated on 10% of the videos, both between the trainers and between the trainers and the groups’ video coders. Cohen's *κ* interobserver reliability 0.85 ± 0.03 (group mean ± s.e. across coders pairs; *sensu* McHugh [89]).

**Table 2.** Ethogram used for data collection with main behavioural categories and operational definitions. (Definitions adapted and modified following previous literature [73–86].)

behavioural category	definition
social play	a sequence of interactive motor behaviours exchanged between two or more individuals including play-specific patterns (e.g. play face, laughter). Typically characterized by role reversals (alternation of attacker/defender), self-handicapping (limiting strength and fighting abilities), graded physical contact and contextual play signals (e.g. play face, brief vocalizations, exaggerated postures) that prevent escalation into conflict. Includes chasing, mock-biting, wrestling and tickling
solitary play	motor, exploratory or manipulative behaviours performed by a single individual in the absence of conspecific partners. Movements include play-specific patterns and are often repetitive, exaggerated or apparently without immediate functional goal (e.g. acrobatics, spinning and object manipulation)
affiliation	peaceful social interactions involving direct contact or prolonged proximity in the absence of tension or avoidance signals. Encompasses grooming, embracing, sitting/standing in contact, huddling and other low-intensity tactile behaviours
agonistic behaviour	threatening interactions directed at a conspecific, from ritualized threat displays (threat postures, aggressive facial expressions and chasing) to high-intensity physical aggression (bites, hits, forceful pushes and kicks). Distinguished from play by the absence of play signals and by the presence of clear anticipatory/aggressive cues

### (b) Operational definitions

Following the approach proposed by Norscia *et al.* [6], we applied a modified version of the post-conflict/matched-control (PC–MC) method, originally formulated to assess post-conflict reunions in animals [90] and later adapted to investigate contagion phenomena such as grooming and yawning [8,91,92]. We defined two conditions: post-play (PP) and MC. In the PP condition, we recorded the onset of a play session—either social or solitary—and used it as the trigger event (time 0, *t*₀) for a 3 min observation period. A 3 min window was selected as a compromise between allowing sufficient time for a response, given that contagion can emerge several minutes after exposure to triggering stimuli, and reducing both the risk of autocorrelation in contagion responses (e.g. in the fourth and fifth minutes, as reported for yawn contagion) and the probability that confounding variables might intervene between stimulus onset and the start of behavioural replication [12,93]. Indeed, play contagion declined to zero once 150 s had elapsed since the perception of the triggering stimulus (supplementary material, figure S1). To avoid confounding effects, the observation began only after at least three uninterrupted minutes without any social interactions. The first individual initiating play was labelled ‘*trigger’*. From *t*₀, in case of play chains (i.e. a sequence of play sessions occurring within 3 min), the last session in the sequence was taken as the trigger event. We then identified as *potential responders* all individuals who, both at *t*₀ and during the 3 min preceding the trigger event: (i) were not directly involved in the ongoing play session; (ii) were not engaged in behaviours that could interfere with the perception or expression of the response (i.e. feeding, sleeping, affiliative or aggressive interactions or other play sessions); and (iii) were present around the trigger event (within 20 m, distinguishing between individuals located shorter or greater than 10 m from the triggering stimulus). These criteria ensured that responders were available to perceive and possibly replicate the observed behaviour.

Each potential responder was observed for 3 min following the onset of the triggering play event to assess whether or not they initiated a new play bout (i.e. a contagion response). We recorded (i) trigger and potential responder identity, sex and age and distance (categorized as a binary factor: ≤ 10 m; 10 m < distance ≤ 20 m); and (ii) trigger play session duration and category (solitary or social). When a potential responder initiated a new bout (thus qualifying as ‘*responder’*), we coded two types of play response depending on whether the responder (i) started a new play session alone or with new players; or (ii) started participating in the existing trigger session (by replacing a previous player or joining them). We also noted down whether the play response category was the same as the trigger play session category (solitary/solitary; social/social) or not (solitary/social or social/solitary). Object play was included and categorized as either social or solitary based on the presence of a partner.

In the MC condition, the same individuals identified as potential responders in the PP session were observed for 3 min at the same time of day (± 1 h) on another suitable day, in the absence of any preceding play activity. MCs were conducted under comparable environmental and social conditions (e.g. similar weather, number of individuals present within visual range), ensuring that the only relevant difference was the absence of a triggering play session.

In both PP and MC conditions, observations were carried out only in the absence of external disturbances (e.g. keepers providing food or cleaning the enclosures), to minimize the possibility that a response behaviour could be elicited by unrelated stimuli rather than by the observed play event.

At the group level, we determined the following measures of social organization and social structure (*sensu* Kappeler & van Schaik [94]): (i) *sex ratio*: proportion of the number of males over the number of females; (ii) *age ratio*: proportion of immature subjects over total subjects (immature + adult subjects); (iii) *group size*: number of individuals in the group; and (iv) *group polarization*: (affiliation − agonistic rates)/(affiliation + agonistic rates). Affiliation and agonistic rates at the group level were calculated as the hourly frequency obtained via the proportion between the number of affiliation or agonistic bouts over the group observation hours.

At the dyadic level, we determined affiliation and agonistic rates between trigger and potential responders (or responder, in case of play response) as the proportion between the number of affiliation and agonistic events and the observation hours of the dyad.

### (c) Statistical elaboration

All statistical analyses were conducted in R v.4.5.0. We ran two blocks of generalized linear mixed models (GLMMs), fitted via the *glmer()* function from the *lme4* package [95]: (i) via GLMM_1,_ on the whole PP-MC dataset (*n* = 4688), we evaluated whether the phenomenon of social play contagion was present and affected by species (i.e. whether play was replicated more in PP than in MC); and (ii) on the PP dataset (*n* = 2344), via GLMMs (two models), we evaluated the possible effect of group identity (GLMM_2_) on the play response likelihood, as well as the effects of play session characteristics, and individual, dyadic and group-level factors (GLMM_3_). Table 3 shows the factors considered for all models. Full models, including fixed effects, were compared to corresponding null models with the same random structure using likelihood ratio tests (*anova(),* test = ‘Chisq’ [96,97]). Individual predictors were tested via the *drop1*() function. Multicollinearity was checked using the variance inflation factor, with all values < 5. Effect sizes were computed using the effectsize package.

**Table 3.** GLMM summary table. (PP refers to post-play condition data; MC to matched-control condition data. Summary of generalized linear mixed models (GLMMs) used to analyse the occurrence of play contagion.)

analysis levels	
model	GLMM_1_
**fixed factors**	condition (PP/MC) × species
**dataset**	PP + MC (*n* = 4688)
**model**	**GLMM_2_**
**fixed factors**	group identity
**dataset**	PP (*n* = 2344)
**model**	**GLMM_3_**
**fixed factors**	responder age + responder sex + trigger age + trigger sex + dyadic affiliation rate (trigger − responder) + dyadic aggression rate (as a control: trigger − responder) + spatial distance (≤10 m vs >10 m) + group size + sex ratio + age ratio + group polarization + play category (solitary vs social) + play bout duration
**dataset**	PP (*n* = 2344)

In all GLMMs, the target variable (play response) was modelled as a binomial distribution (presence/absence). Model GLMM_1_ included the group as a random factor, whereas all other models included the responder identity as a random factor. For GLMM_2_ that included group identity as a fixed factor, significant differences between the full and corresponding null models (likelihood ratio test: $\chi^{2}$, d.f., *p* < 0.05) were followed up with pairwise comparisons of estimated marginal means. Estimated marginal means were computed with the *emmeans* package [98], and pairwise comparisons were adjusted using Tukey's method via the *multcomp* package [99] to identify which specific groups differed from each other. Although some methodological studies do not discuss GLMMs explicitly, the same method could be applied to GLMMs because estimated marginal means are model-based predictions and multiple-comparison adjustments are performed on contrasts derived from the fitted model [99,100]. Furthermore, to ensure the robustness of the full versus null model comparisons, we performed a bootstrap with 1000 iterations for all GLMMs via the *PBmodcomp()* function of the *pbkrtest* package [101]. This approach provides a more reliable estimation of *p*-values by simulating a null distribution specific to the model structure, especially when asymptotic assumptions for the likelihood ratio test may not hold.

## 3. Results

### (a) Play contagion presence

**Figure 2.**
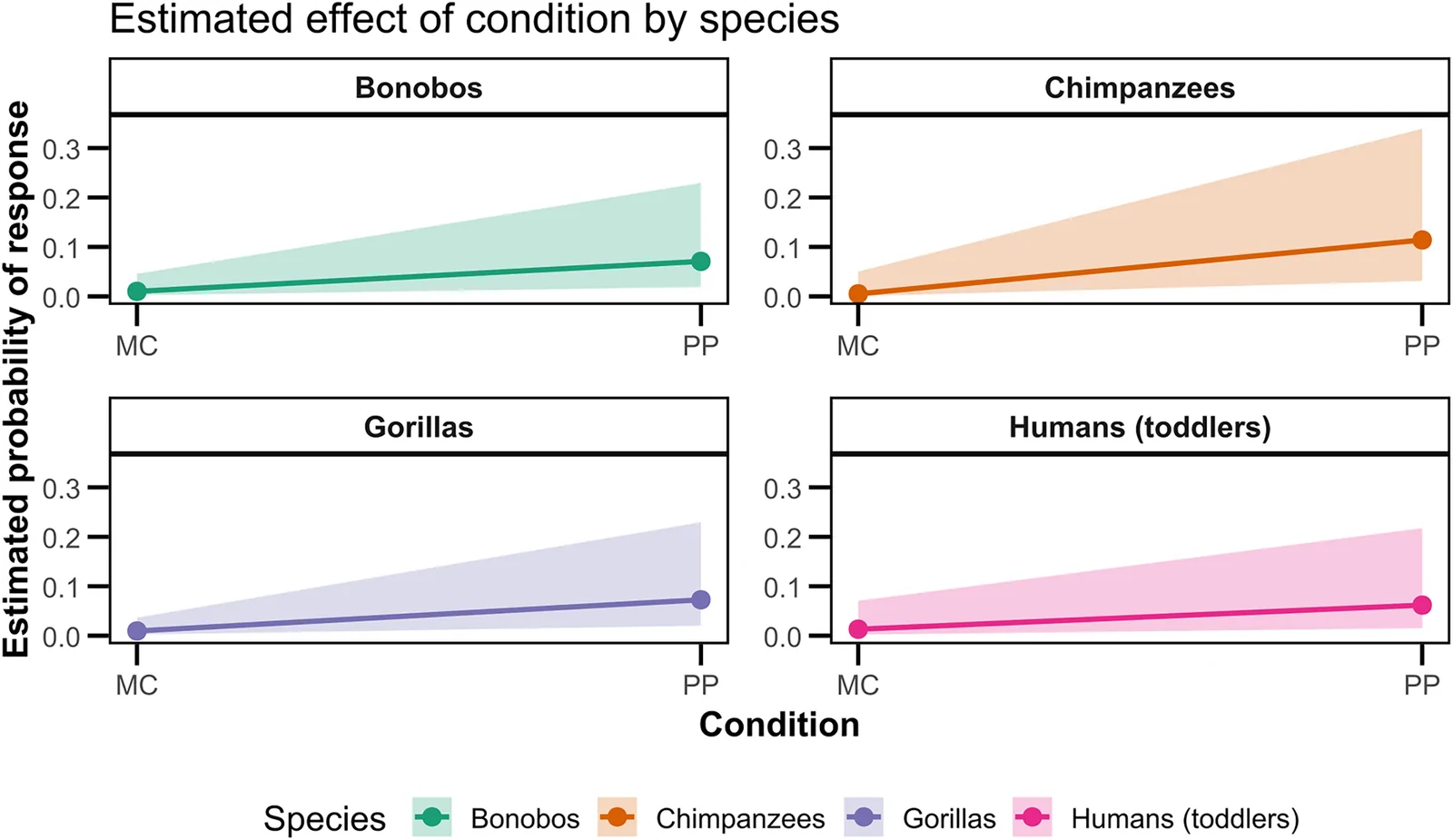
Effect plots showing the estimated probability of response (*y*-axis) for bonobos, chimpanzees, gorillas and humans (toddlers), based on the experimental condition (*x*-axis: MC and PP). The 95% confidence interval is represented by the shaded band.

GLMM_1_ (*n* = 4688) was carried out to verify the presence of play contagion phenomenon (play response observed more in PP than in MC). The full model—which included all fixed factors (condition PP/MC, species and their combination)—and the null model including only the random factor (group identity) significantly differed (likelihood ratio test: $\chi_{7}^{2}$ = 253.26; *p* < 0.001, significance confirmed by bootstrap with 1000 iterations: *p* < 0.001; see table 4 and figure 2). Because at least one predictor was significant, we applied the *drop1()* procedure, which showed that only the factor condition predicted play response (*p* < 0.001; figure 2): play was more likely after a play session (PP) than in control observations (MC). Neither species nor its interaction with condition was significant (table 4). Therefore, the observed increase in play occurrence following a social trigger confirms the presence of play contagion across all four study species.

**Table 4.** GLMM results table. (Note: significant effects are shown in bold. Random factors: group identity (GLMM_1_); trigger × responder combination (GLMM_2–4_), to account for repeated observations. Detailed results of the GLMMs. For each model, the number of observations (*n*_cases_), full versus null model comparison (χ², d.f. and *p*), fixed-effect estimates (estimate ± s.e.m.), 95% confidence intervals (CI_95_), effect sizes, *z*- and *p*-values are reported.)

predictors	estimate	s.e.m.	CI_95_	effect size	*z*	*p*-value
GLMM_1_. *n* = 4688; *full vs. null model*: $\chi_{7}^{2}$ = 253.26; *p* < 0.001						
(intercept)	−4.585	0.788	[−6.13, −3.04]	^a^	^a^	^a^
condition [PP]^b^	2.010	0.443	[1.14, 2.88]	2.01	4.542	**<0.001**
species [chimpanzees]^b^	−0.696	1.429	[−3.50, 2.11]	−0.70	−0.487	0.626
species [gorilla]^b^	−0.057	1.049	[−2.11, 2.00]	−0.06	−0.054	0.957
species [human] ^b^	0.262	1.189	[−2.07, 2.59]	0.26	0.221	0.825
condition:species	1.220	1.106	[−0.95, 3.39]	1.22	1.104	0.270
condition:species	0.085	0.478	[−0.85, 1.02]	0.08	0.177	0.859
condition:species	−0.407	0.773	[−1.92, 1.11]	−0.41	−0.527	0.598
GLMM_2_. *n* = 2344; *full vs. null model*: $\chi_{7}^{2}$ = 15.86; *p* = 0.026						
(intercept)	−2.599	0.602	[−3.74, −1.38]	^a^	^a^	^a^
BONO_2^b^	−1.802	0.851	[−3.47, −0.13]	−1.80	−2.118	**0.034**
CHIMP_1^b^	−0.318	0.994	[−2.27, 1.63]	−0.32	−0.319	0.749
CHIMP_2^b^	0.294	0.915	[−1.50, 2.09]	0.29	0.321	0.748
GOR_1^b^	0.372	0.789	[−1.17, 1.92]	0.37	0.472	0.637
GOR_2^b^	−2.735	0.930	[−4.56, −0.91]	−2.74	−2.942	**0.003**
HUM_1^b^	−0.593	0.794	[−2.15, 0.96]	−0.59	−0.747	0.455
HUM_2^b^	−1.071	1.055	[−3.14, 1.00]	−1.07	−1.016	0.310
GLMM_3_. *n* = 2344; *full vs. null model*: $\chi_{13}^{2}$ = 94.10; *p* < 0.001						
(intercept)	−3.021	0.240	[−3.49, −2.55]	^a^	^a^	^a^
trigger age	−0.043	0.090	[−0.22, 0.13]	−0.04	−0.472	0.637
trigger sex [1]^b^	0.088	0.236	[−0.37, 0.55]	0.09	0.372	0.710
responder age	−2.168	0.286	[−2.73, −1.61]	−2.17	−7.593	**<0.001**
responder sex [1]^b^	−0.849	0.205	[−1.25, −0.45]	−0.85	−4.148	**<0.001**
dyadic affiliation rate (trigger − responder)	0.135	0.134	[−0.13, 0.40]	0.14	1.009	0.313
dyadic aggression rate (trigger − responder)	−0.144	0.097	[−0.33, 0.05]	−0.14	−1.484	0.138
group size	−0.159	0.193	[−0.54, 0.22]	−0.16	−0.824	0.410
sex ratio	0.994	0.192	[0.62, 1.37]	0.99	5.173	**< 0.001**
age ratio	−0.411	0.157	[−0.72, −0.10]	−0.41	−2.624	**0.009**
polarization	1.296	0.180	[0.94, 1.65]	1.30	7.191	**<0.001**
spatial distance (≤10 m vs >10 m) [1]	−0.721	0.270	[−1.25, −0.19]	−0.72	−2.675	**0.007**
play category	−0.093	0.174	[−0.43, 0.25]	−0.09	−0.531	0.595
play bout duration	−0.039	0.086	[−0.21, 0.13]	−0.04	−0.450	0.652

^a^ Intercepts are not shown, as they have no meaningful interpretation.

^b^ Dummy-coded variables; reference categories were: condition = MC; species = bonobo; group identity = BONO_1; sex = male; play category (solitary versus social) = 0 (social play); spatial distance (≤ 10 m versus > 10 m) = 1 (> 10 m).

### (b) Factors influencing play contagion occurrence

**Figure 3.**
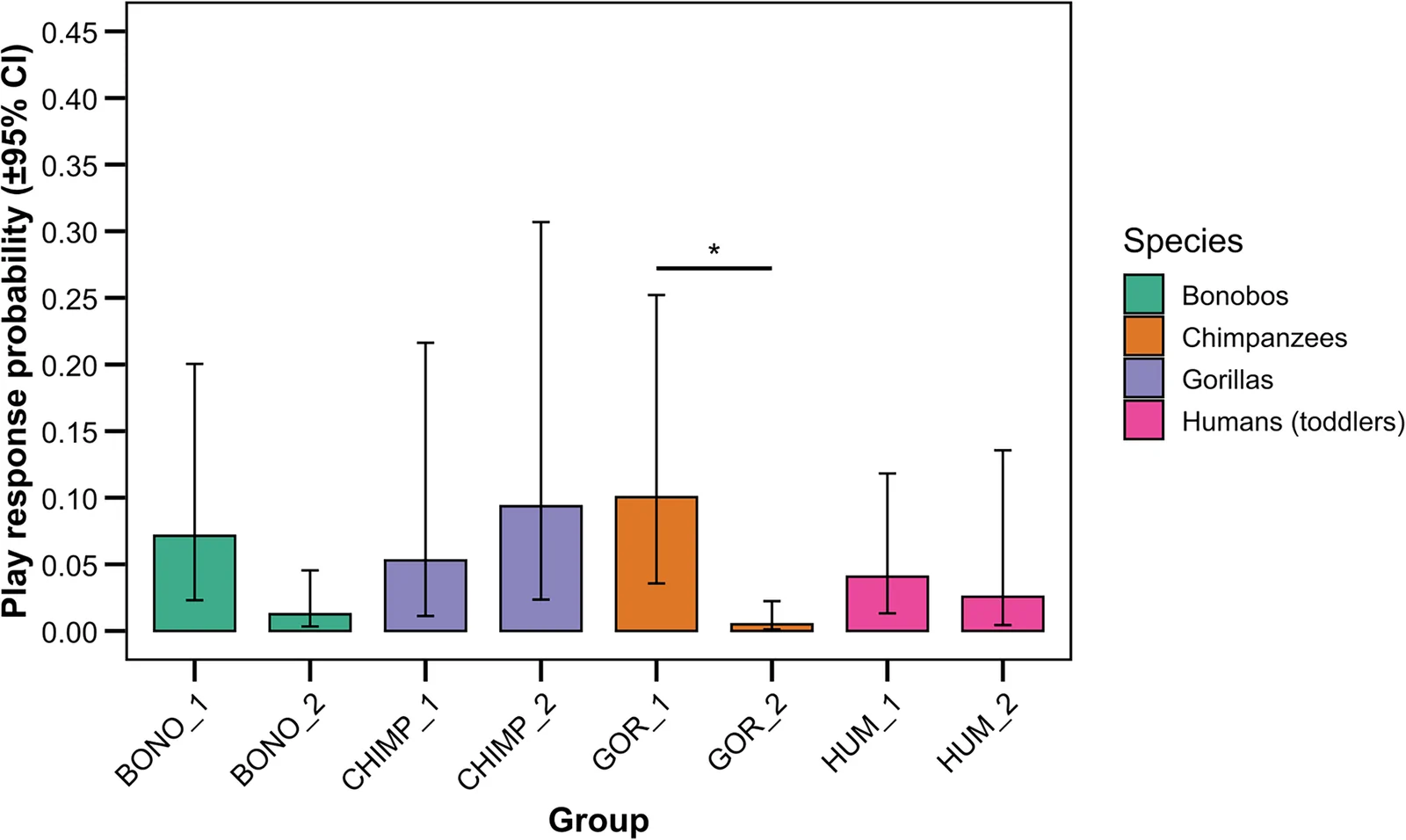
The bar plot shows the estimated mean play response probability (*y*-axis) for the different groups (*x*-axis) within each of the four species (bonobos, chimpanzees, gorillas and humans). The vertical error bars represent the 95% confidence interval. *p < 0.05.

GLMM_2_ (*n* = 2344) was carried out to test whether social group identity influenced the probability of contagion within PP observations. The full model—which included group identity as fixed factor and responder identity as random effect—significantly differed from the corresponding null model (likelihood ratio test: $\chi_{7}^{2}$ = 15.86; *p* = 0.026; significance confirmed by bootstrap with 1000 iterations: *p* = 0.041; see table 4). The *drop1()* procedure showed that two groups, BONO_2 (*p* = 0.034; table 4) and GOR_2 (*p* = 0.003; table 4), had significantly lower contagion rates compared with the reference group (BONO_1). No other group effect was significant. However, pairwise comparisons (Tukey test) showed that only GOR_2 exhibited a significantly lower contagion probability than GOR_1 (*p* = 0.018; figure 3; supplementary material, table S2 and figure S2). All other pairwise comparisons were non-significant (supplementary material, table S2).

**Figure 4.**
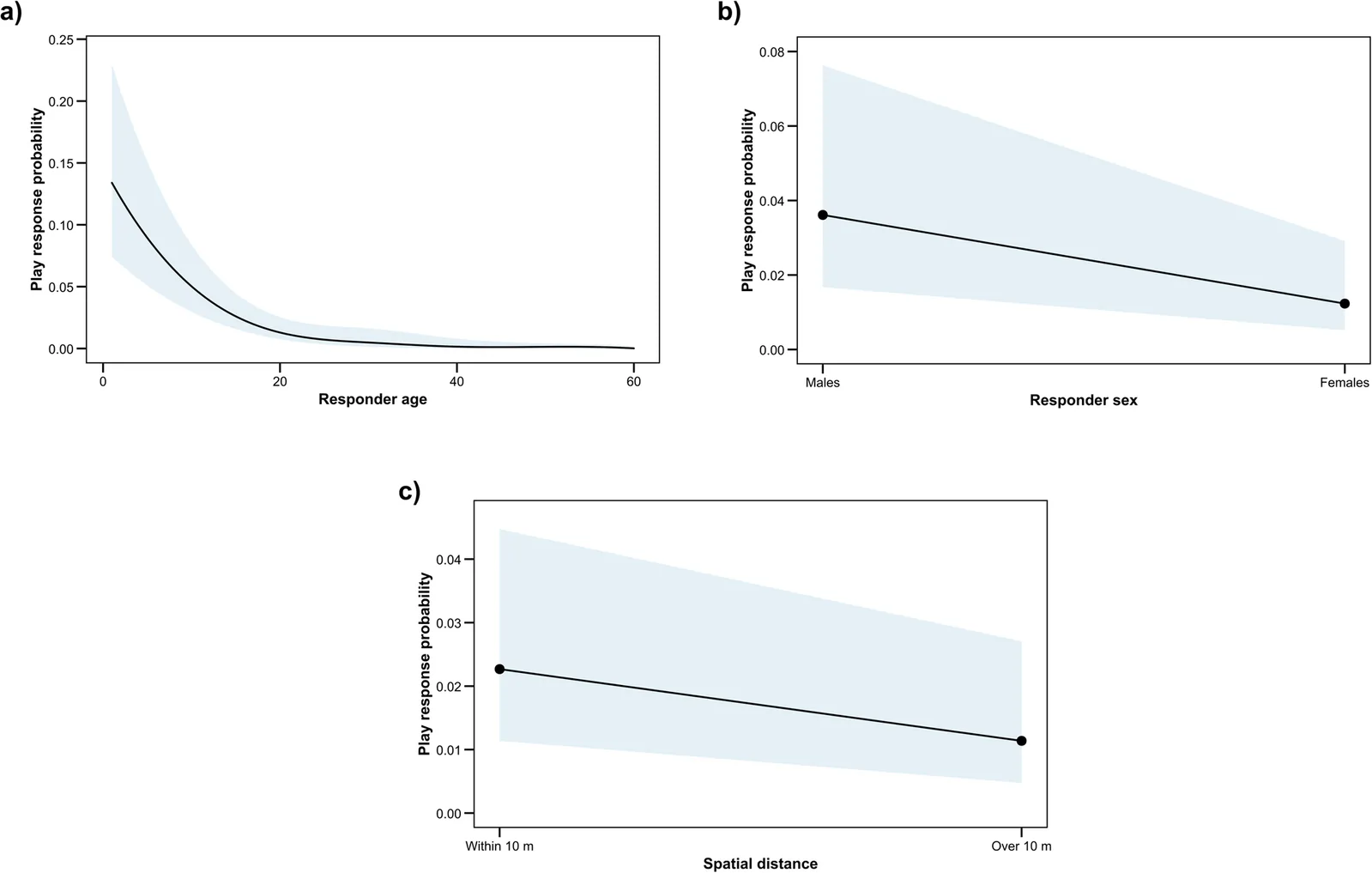
Effect plots showing the estimated probability of play response (*y*-axis) based on three significant predictors: (a) responder age, (b) responder sex, and (c) spatial distance. The 95% confidence interval is represented by the shaded band.

**Figure 5.**
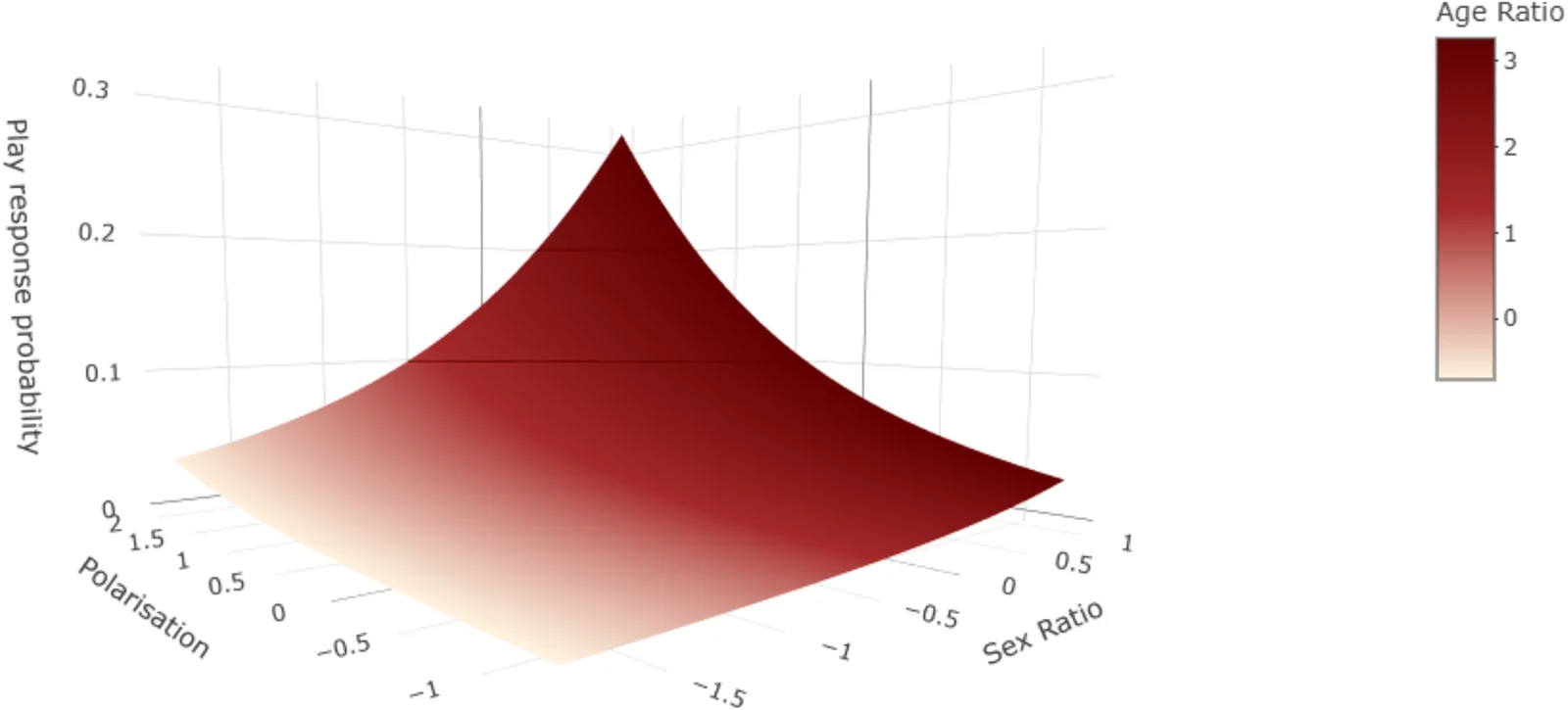
Three-dimensional response surface plot illustrating the play response probability (*z*-axis) as a function of the interaction between group-level factors. The surface height represents the predicted probability of play contagion, while the *x*-axis and *y*-axis represent the **sex ratio** (proportion of females) and **group polarization**, respectively. The colour gradient (from cream to amaranth) maps the effect of the **age ratio**, providing a four-dimensional representation of the socio-demographic interaction.

GLMM_3_ (*n* = 2344) evaluated the influence of play session features, individual/dyadic factors and group-level variables on the probability of play contagion. The full model significantly differed from the corresponding null model (likelihood ratio test: $\chi_{13}^{2}$ = 94.10; *p* < 0.001; significance confirmed by bootstrap with 1000 iterations: *p* < 0.001; table 4). Regarding session characteristics, neither play category nor play bout duration significantly affected the likelihood of play being replicated. In terms of individual and dyadic predictors, *drop1()* revealed that younger individuals were more likely to join play (*p* < 0.001; figure 4a), males responded more often than females (*p* = 0.016; figure 4b), and a distance ≤ 10 m was associated with an increased probability of contagion (*p* = 0.05; figure 4c). No effects were found for trigger-related variables (age/sex) or dyadic relationship measures (affiliation and aggression rates). Finally, concerning group structural/organizational measures, *drop1()* identified sex ratio (*p* < 0.001; figure 5), age ratio (*p* = 0.009; figure 5) and group polarization (*p* < 0.001; figure 5) as significant positive predictors of play response, whereas group size was not significant.

## 4. Discussion

We found the presence of play contagion across all social hominid species, with play occurring significantly more often after a play session (PP condition) than in the absence of it (MC condition) (*prediction 1* confirmed; table 4 and figure 2). Play session features— type (solitary/social) and duration—had no effect on play contagion occurrence (*prediction 2* confirmed; table 4). At the individual and dyadic level, males and younger individuals were most likely to show play contagion (*prediction 3a* confirmed; table 4 and figure 4a,b), whereas among dyadic variables, spatial proximity, but not affiliation and aggression rates, showed a significant effect in enhancing contagion (*prediction 3b* partially confirmed; table 4 and figure 4c). Contagion levels varied across social groups but a significant difference was observed only between the two gorilla groups, whereas no other pairwise comparisons were statistically significant (*prediction 4a* partially confirmed; table 4 and figure 5). Among demographic variables, contagion increased in groups with the highest sex ratio (male-to-female) and the highest age ratio, whereas group size had no significant effect (*prediction 4b* partially confirmed; table 4 and figure 5). Regarding social dynamics, higher affiliative relative to agonistic interactions (social polarization) was associated with higher play contagion occurrence *prediction 4c* confirmed; table 4 and figure 5).

Previous research has largely focused on single groups or cohorts and on individual and dyadic variables in behavioural contagion—mainly of yawning but also scratching and grooming (for review see [2,11])—while cross-species comparative evidence on play contagion is virtually absent. In the present investigation, we demonstrated the presence of play contagion across all social hominid species, for the cohorts included in the study, reporting it for the first time in bonobos and gorillas and confirming previous evidence in humans [9]—where it is here quantitatively assessed—and in chimpanzees [7]. Given the adaptive value of play in fostering social tolerance, cooperation and emotional attunement [58,59], its contagious nature could have contributed to the evolution of social dynamics in group-living hominids. The presence of play contagion in all the cohorts of the species considered—without appreciable interspecific differences—is not trivial, especially when compared to other forms of behavioural contagion. For instance, among apes, yawn contagion has been documented in bonobos, chimpanzees and humans [3,8,19,21,25–27,29,62,65], but not in lowland gorillas [102], although it has been reported in orangutans [103], the least socially cohesive among hominids. However, some behavioural contagion phenomena may become observable under certain social conditions and not under others, an issue to be considered in future studies.

The lack of effect of play session features—whether the triggering play session was solitary or social or of different duration—is consistent with the idea that play contagion can be elicited by both social and non-social play cues, may occur independently of play type and can unfold over variable time windows from the onset of the triggering event [9,40,44]. Moreover, in behavioural contagion, what is replicated is typically the same general behavioural category, while the specific pattern may vary considerably [12].

At the individual level, age and sex of the potential play responder emerged as significant modulators of play contagion. Males and younger individuals were most likely to exhibit contagion, a pattern that aligns with the role of play as a context for competitive yet non-aggressive interactions, motor training and the general age-dependence of play behaviour [6,39,55–59,66–69]. Juveniles often display higher responsiveness to social stimuli and greater motor flexibility [104,105], which may increase the probability of replicating others’ play behaviour through perception–action mechanisms. A similar trend has been documented in African elephants (*L. africana*); play contagion also tended to peak in younger age classes [6]. In the context of play, males may be more prone to behavioural contagion because they use play to test strength and compete for hierarchical status while maintaining affiliative relationships [52,106–108]; however, this may vary in female-centred societies, such as bonobos, where status is not strictly based on overt fighting or intense aggression. At the dyadic level, spatial proximity significantly increased the likelihood of contagion, as predicted by the perception–action model [3,4], which posits that the probability of contagion depends on the salience and detectability of the triggering stimulus, which is enhanced by physical closeness. A similar result was found for play contagion in African elephants (*L. africana* [6]) and for the contagion of other behaviours (e.g. yawn contagion in lemur *I. indri* [29]). The lack of a significant role for affiliation on play contagion can be related to behavioural contagion as a context-dependent phenomenon [2]. For different behaviours, the relationship between their contagion and social closeness has been reported as positive, negative or absent. In naturalistic studies, positive effects have been reported for grooming contagion in chimpanzees [7], scratching contagion in Tibetan macaques (*Macaca thibetana* [5]), and yawn contagion in geladas, Indri lemur and humans [26,26,29,109–111]. Conversely, negative effects emerged for yawn contagion in toddlers [8] and scratching contagion in orangutans (*Pongo pygmaeus* [112]). Finally, no significant effect of social closeness was found for grooming contagion in rhesus macaques [92], yawn contagion in chimpanzees [62] and spider monkeys (*Ateles geoffroyi* [113]) or yawn and scratching contagion in ruffed lemurs (*V. variegata* and *V. rubra* [27]). In different bonobo groups, the relation between social closeness and yawn contagion was found positive [19], negative [28] or absent [21]. Regarding play contagion, Sandars *et al.*[7] found no effect of social closeness on play contagion in chimpanzees, whereas a positive effect was found by Norscia *et al.* [6] in African elephants (*L. africana*). These pieces of evidence suggest that the modulation of contagion by affiliation varies according to the behavioural domain, group and social contexts. In the case of play contagion, the shift in effect of social closeness can depend on whether play serves as a means of testing social boundaries or reinforcing affiliative bonds.

Moving to the group level, play contagion did not differ considerably among groups, except for two gorilla groups, possibly owing to the higher mean age of the gorillas at La Vallée des Singes (22.6 years) compared to those at Beauval (11.67 years). We cannot exclude the influence of management conditions, which were similar but not identical and could also explain why the model and pairwise comparisons did not reveal clear or consistent differences among groups. Moreover, groups may converge in some aspects of their composition and social dynamics but not in others, which can have contributed to blurring differences among them. Indeed, an important outcome of this study is that, beyond individual and dyadic variables, demographic and social dynamic factors—corresponding to aspects of social organization and social structure, respectively (*sensu* Kappeler & van Schaik [94])—also affected play contagion. A higher age ratio of immature individuals to adults enhanced play contagion, as expected, given that play is a ubiquitous trait of immature individuals and peaks within the weaning period. Not only immature individuals play more but they may also be more likely to be affected by the playful mood of others [6,7,38,39,47,50,57]. Group size did not affect contagion probability, probably because behavioural contagion primarily involves individuals belonging to a subgroup spatially close to the trigger stimulus, that is those capable of perceiving it. As discussed above, perception is a key prerequisite for the activation of perception–action coupling and the ensuing motor resonance. Moreover, not all individuals are equally susceptible to behavioural contagion, as shown for yawn contagion in bonobos and humans [21,26,111], meaning that even in larger groups, contagion may remain confined to a subset of responsive individuals. Play contagion increased with a higher male-to-female ratio and in groups that were socially polarized towards affiliation rather than aggression, supporting the view that behavioural contagion functions as a group-level coordination and cohesive process [2,30]. The positive association between contagion and a male-biased sex ratio may stem from what we observed at the individual level—namely that males were more susceptible to contagion, probably because they use play for motor training and physical and social assessment of future competitors [52,106–108]. In this respect, play contagion may contribute to regulating social tension and stabilizing hierarchical relationships through non-aggressive interactions. Finally, the effect of social polarization towards affiliation on play contagion indicates that this phenomenon is favoured by cohesive rather than disruptive group behaviour, which is consistent with play being linked with social tolerance under certain circumstances [6,58,59].

Although this investigation includes the largest sample ever examined in relation to behavioural contagion in primates (eight groups; two per social hominid species), the possibility of generalizing the results on the group-level variables influencing play contagion depends on enlarging the sample in future studies. Moreover, our data are silent on whether such tolerance emerges from or contributes to the emergence of play contagion. Complex behavioural contagion phenomena and their correlates may be connected through nonlinear dynamics [114], which future investigation is welcome to delve into. Additionally, future studies considering cohorts beyond WEIRD populations (Western, Educated, Industrialized, Rich, and Democratic) and taking into account the STRANGE framework (Social background, Trappability and self-selection, Rearing history, Acclimation and habituation, Natural changes in responsiveness, Genetic make-up, and Experience) may reveal variation in play contagion dynamics that could help trace the complex profile of this phenomenon. In this respect, operationalizing some of these aspects into measurable variables may represent an important challenge for future research. Finally, in an ape–human evolutionary perspective, the inclusion of orangutans would be particularly valuable. Although typically semi-solitary, orangutans show flexible sociality, being more gregarious in some wild populations [115], show conflict resolution [60] and display diverse forms of play [116], as well as behavioural contagion, namely yawning [103] and scratching contagion [112]. Their present social organization may reflect ecological constraints linked to resource distribution, and higher sociality has been hypothesized for the evolutionary past [117]. Thus, we expect play contagion to be present in orangutans, even if such processes may be less frequent or more difficult to observe under their typical social system.

In conclusion, although extending the findings beyond the cohort considered here requires caution, this study points to the possibility that play contagion might be a shared phenomenon across all social hominid species and that it may involve common modulators operating both within groups and at demographic and social dynamic levels. Thus, we may hypothesize that play contagion, together with its shared modulators, has contributed, since the last common ancestor of social hominids (who lived approx. 7.2–11 Ma; [118]), to the diffusion of non-aggressive interactions—and possibly their underlying emotions—with implications for the evolution of human social dynamics.

## Supplementary Material



## Data Availability

Data (in a calculation sheet) are included as supplementary material (Data_S1). Supplementary material is available online [119].

## Theme

One contribution of 19 to a theme issue ‘Mechanisms, development, phylogeny and functions of emotional expressions’.
